# The crossed frontal aslant tract: A possible pathway involved in the recovery of supplementary motor area syndrome

**DOI:** 10.1002/brb3.926

**Published:** 2018-02-05

**Authors:** Cordell M. Baker, Joshua D. Burks, Robert G. Briggs, Adam D. Smitherman, Chad A. Glenn, Andrew K. Conner, Dee H. Wu, Michael E. Sughrue

**Affiliations:** ^1^ Department of Neurosurgery University of Oklahoma Health Sciences Center Oklahoma City OK USA; ^2^ Department of Radiological Sciences University of Oklahoma Health Sciences Center Oklahoma City OK USA

**Keywords:** supplementary motor area syndrome, tractography, white matter connectivity

## Abstract

**Introduction:**

Supplementary motor area (SMA) syndrome is a constellation of temporary symptoms that may occur following tumors of the frontal lobe. Affected patients develop akinesia and mutism but often recover within weeks to months. With our own case examples and with correlations to fiber tracking validated by gross anatomical dissection as ground truth, we describe a white matter pathway through which recovery may occur.

**Methods:**

Diffusion spectrum imaging from the Human Connectome Project was used for tractography analysis. SMA outflow tracts were mapped in both hemispheres using a predefined seeding region. Postmortem dissections of 10 cadaveric brains were performed using a modified Klingler technique to verify the tractography results.

**Results:**

Two cases were identified in our clinical records in which patients sustained permanent SMA syndrome after complete disconnection of the SMA and corpus callosum (CC). After investigating the postoperative anatomy of these resections, we identified a pattern of nonhomologous connections through the CC connecting the premotor area to the contralateral premotor and SMAs. The transcallosal fibers have projections from the previously described frontal aslant tract (FAT) and thus, we have termed this path the “crossed FAT.”

**Conclusions:**

We hypothesize that this newly described tract may facilitate recovery from SMA syndrome by maintaining interhemispheric connectivity through the supplementary motor and premotor areas.

## 1 INTRODUCTION

The supplementary motor area (SMA) and pre‐SMA are involved in the initiation, learning, and planning of complex motor tasks. Tumors involving the SMA are not an infrequent occurrence as previous data suggest that 10% of de novo glioblastomas and 27% of WHO grade II gliomas occur in this region (Duffau & Capelle, 2004). Patients undergoing resection involving the SMA can develop SMA syndrome, characterized by transitory hemiakinesia with preservation of muscle tone (Tate, Kim, Chang, Polley, & Berger, 2011; Vassal et al., 2017; Vergani et al., 2014). SMA syndrome may also lead to mutism and speech hesitancy when lesions involve the dominant hemisphere (Ryu, Chun, & You, 2013). In patients with SMA syndrome, the brain shows a remarkable capacity for compensation after unilateral lesions as roughly 90% of patients recover from SMA insult over the course of weeks to months (Kim et al., 2013; Rosenberg et al., 2010).

The cause of postoperative SMA syndrome, along with the mechanism of recovery, has been investigated in the past but has yielded limited information (Rosenberg et al., 2008; Tate et al., 2011; Vassal et al., 2017). Functional MRI (fMRI) studies in those who have recovered from SMA syndrome demonstrated an increase in connectivity between ipsilateral primary sensorimotor cortex and contralateral SMA and premotor areas (PMAs) compared to preoperative values (Vassal et al., 2017). This suggests that SMA recovery may rely at least in part, on a compensatory mechanism that involves connections to the contralateral SMA and PMA.

Earlier work with tractography has revealed a novel short fiber tract connecting the posterior portion of the inferior frontal gyrus to the ipsilateral SMA and pre‐SMA (Catani, Dell'acqua, & Vergani, 2012; Catani et al., 2013; Ford, McGregor, Case, Crosson, & White, 2010). Catani et al. termed this tract the frontal aslant tract (FAT) (Catani et al., 2012). In this paper, we describe a path of connectivity between the FAT and contralateral PMA, through the corpus callosum (CC). These transcallosal fibers appear to originate with the FAT, and thus, we have termed this tract the “crossed FAT.” Others have described transcallosal SMA fascicles previously but made no mention of its connection with the PMA or its association with the FAT (Lemaire et al., 2013; Vergani et al., 2014). Furthermore, no one has illustrated how these fibers may assist in the recovery of SMA syndrome.

In this paper, we present two cases of patients who underwent surgery for tumors involving the SMA. Both patients developed SMA syndrome that did not improve on long‐term follow‐up. Careful analysis of the anatomy of these resections led us to hypothesize that the disruption of contralateral connections prevented SMA syndrome recovery. We performed anatomic studies and present evidence of white matter (WM) projections from the FAT that connect the PMA and contralateral PMA and SMA. While ethical considerations make it difficult to prove that this tract is necessary in order to improve from SMA syndrome, we suggest that taken together, this data provide a plausible mechanism for the recovery of SMA syndrome.

## 2 METHODS

### 2.1 Anatomic boundaries of the SMA for ROI selection

In this study, we will focus on a gyrus‐based description of the SMA to aid in surgical conceptualization, although we acknowledge that this area can be variable across individuals (Ribas, 2010). The SMA was taken to be the caudal region of the frontal lobe that resides within the interhemispheric fissure, just anterior to the primary motor cortex. The precentral sulcus, immediately anterior to the precentral gyrus, comprises the posterior border of the SMA. The anterior border was designated as 5 cm anterior to the precentral sulcus, as described by others (Vergani et al., 2014). The SMA is separated from the pre‐SMA by the vertical line of the anterior commissure. This line intersects the anterior commissure and is perpendicular to the anterior commissure–posterior commissure plane (Lehericy et al., 2004). The inferomedial limit of the SMA is taken to be the cingulate sulcus, and the inferolateral boundary taken to be the superior frontal sulcus (Vergani et al., 2014).

### 2.2 DSI tractography

Publicly available imaging data from the Human Connectome Project (HCP, RRID:SCR_008749) were obtained for this study from the HCP database (http://humanconnectome.org, release Q3). A multishell diffusion scheme was used, and the b‐values were 990, 1,985, and 1,980 s/mm^2^. Each b‐value was sampled in 90 directions. The in‐plane resolution was 1.25 mm. The slice thickness was 1.25 mm. The diffusion data were reconstructed using generalized q‐sampling imaging with a diffusion sampling length ratio of 1.25 (Yeh, Wedeen, & Tseng, 2010).

We performed brain registration to Montreal Neurologic Institute (MNI) space, wherein imaging is warped to fit a standardized brain model for comparison between subjects (Ardekani et al., 2011). Tractography was performed in DSI studio (Carnegie‐Melon, RRID:SCR_009557) using two predefined regions of interest (ROIs) to isolate single tracts (Martino et al., 2011; Yeh et al., 2010). Voxels within each ROI were automatically traced with randomized seeding of the voxel and/or tract with a maximum angular threshold of 45 degrees. When a voxel was approached with no tract direction or a direction change >45 degrees, the tract was halted. Tractography was stopped after reaching a length of 450 mm. In some instances, exclusion ROIs were placed to exclude obviously spurious tracts that were not involved in the network of interest. ROIs were placed manually, and their position verified by an attending neurosurgeon comfortable with the cortical anatomy visible on imaging.

### 2.3 Postmortem dissection

The purpose of the postmortem dissections was to verify the existence of the tractography results as ground truth. Postmortem dissections were performed in 10 hemispheres using a modified Klingler technique (Silva & Andrade, 2016). The specimens used for this study were obtained from our institution's Willed Body Program with approval of the state's anatomical board. Relevant cortical areas were identified first. Starting superficially, they were then peeled back to reveal white matter areas of interest. Care was taken to leave cortical areas corresponding to white matter tracts of interest intact in order to preserve their relationship. Tracts were dissected with blunt instruments to avoid disrupting the natural tract anatomy.

### 2.4 Selected cases

Two patients who underwent glioma resection by the senior author are presented as their lack of recovery may be dependent on connections between SMA and PMA callosal fibers. The patients had gliomas in the left frontoparietal region within the anatomic area of the SMA. The ventricle was widely entered in both cases suggesting that the CC was cut. Both patients sustained permanent postoperative deficits. Patients were evaluated by the senior author, and SMA syndrome was defined as immediate postoperative akinesia more prominent contralateral to the lesioned side, with or without speech hesitancy or mutism (Laplane, Talairach, Meininger, Bancaud, & Orgogozo, 1977; Potgieser, de Jong, Wagemakers, Hoving, & Groen, 2014). Permanent SMA syndrome was defined as continued hemiplegia or hemiparesis with or without speech difficulty on long‐term follow‐up (Bannur & Rajshekhar, 2000; Ibe et al., 2016). Of note, these patients were identified retrospectively and were the only patients in the last 5 years who did not show improvement from SMA syndrome on follow‐up visits. Patient data were included in this study with approval from our institutional review board (IRB #3199).

## 3 RESULTS

### 3.1 Anatomical evidence for the existence of the crossed FAT

**Figure 1:**
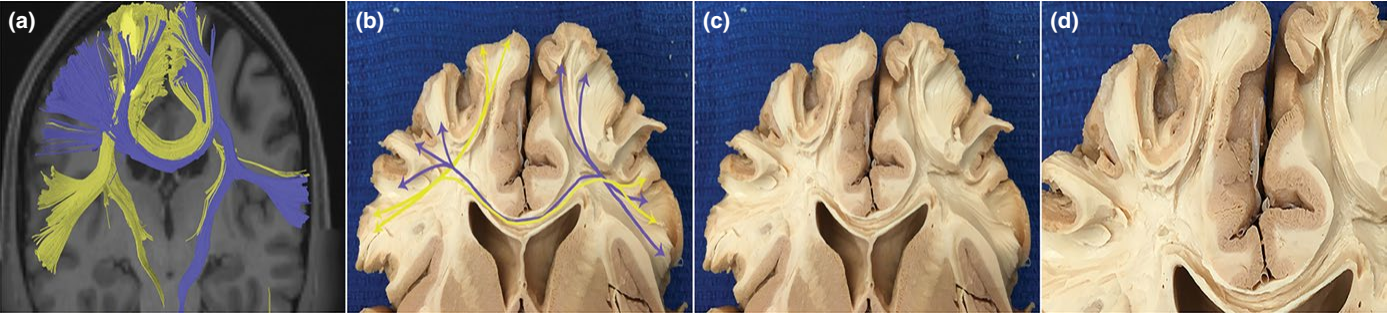
Interhemispheric white matter connections of the supplementary motor area (SMA) and premotor area (PMA) through the corpus callosum (CC). (a) Tractography illustrating white matter path from the frontal aslant tract (FAT) to contralateral SMA and PMAs. Fibers in blue are originating from the left hemisphere; yellow fibers are originating from the right. (b) Gross anatomical dissection of fiber bundles, tracts are similar to the ones illustrated in (a). Fiber tracts (blue and yellow arrows) originate from the FAT and course through the CC to end at the contralateral PMA. (c) Same image as (b) without markings. (d) Closer view of white matter bundles exiting the CC toward left and right PMAs

We encountered contralateral connections of areas involved in SMA syndrome. These findings are provided from our tractography analysis and grounded in 10 gross anatomic dissections. Starting at the medial aspect of the cerebrum at the SMA, the white matter fibers course caudally and divide into two tracts just superolateral to the CC. One tract continues caudally around the lateral ventricle before splitting again and traveling toward the inferior frontal gyrus and striatum. The other tract superolateral to the CC turns medially, traversing the anterior aspect of the CC to the other hemisphere with the fiber bundles ending at the contralateral PMA. Both right and left SMA were shown to have fibers bundles traversing the CC. Comparison between tractography and gross anatomical dissection is shown in Figure 1. Tractography and gross dissection both show transcallosal fibers exiting the CC and traveling toward the PMA. All 10 of the cadaveric brain dissections demonstrated these tracts consistently.

### 3.2 Case evidence for the importance of CC connections in SMA recovery

#### 3.2.1 Case 1

**Figure 2:**
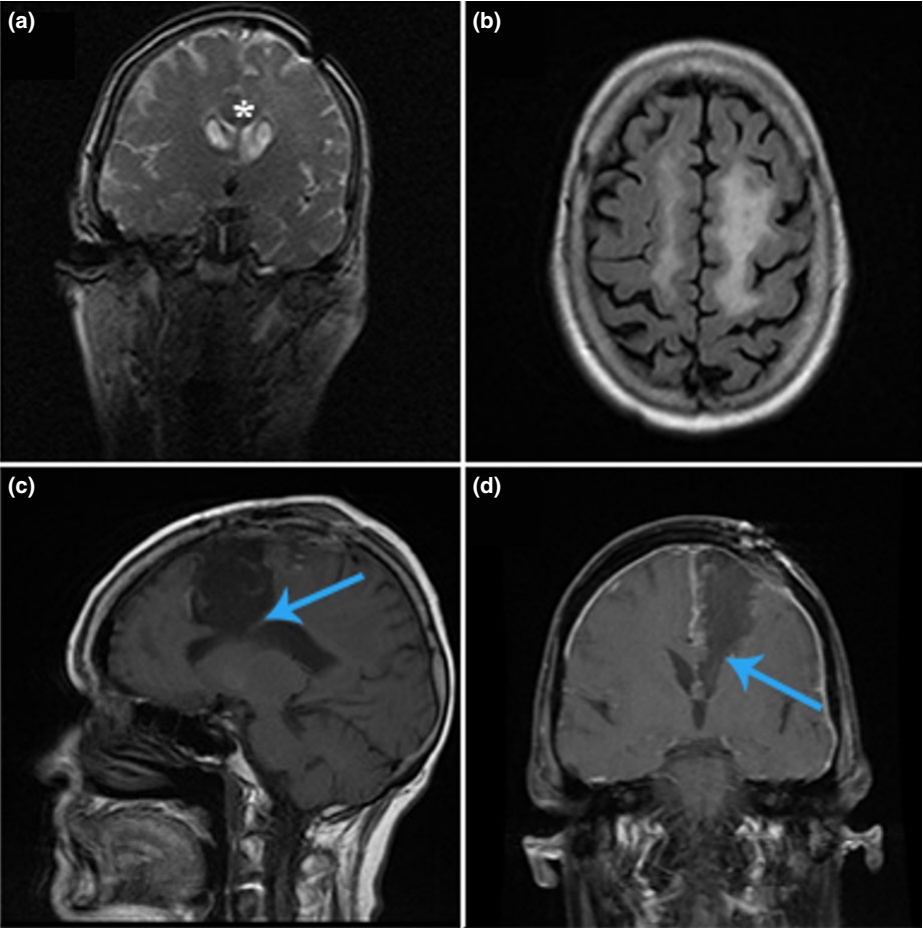
Permanent supplementary motor area (SMA) syndrome, case 1. Patient with a low‐grade glioma of the left frontoparietal region, hyperintensities seen on coronal T2 (a) and on sagittal T2 FLAIR (b) imaging. Postresection of tumor revealing dissection through the SMA into the corpus callosum (CC) on T1 with (d) and without (c) contrast (blue arrows). Asterisks in (a) designate location of CC

A 62‐year‐old woman diagnosed with a low‐grade glioma of the left frontoparietal region presented with refractory seizures but no other deficits. She underwent an awake craniotomy for tumor resection. Immediately postoperatively, the patient was found to have mutism and was unable to move the right side of her body. The patient sustained these deficits permanently with no improvement at 3 years. Preoperative and postoperative imaging of the resection is illustrated in Figure 2. The postoperative scan shows removal of the SMA and portions of the CC.

#### 3.2.2 Case 2

**Figure 3:**
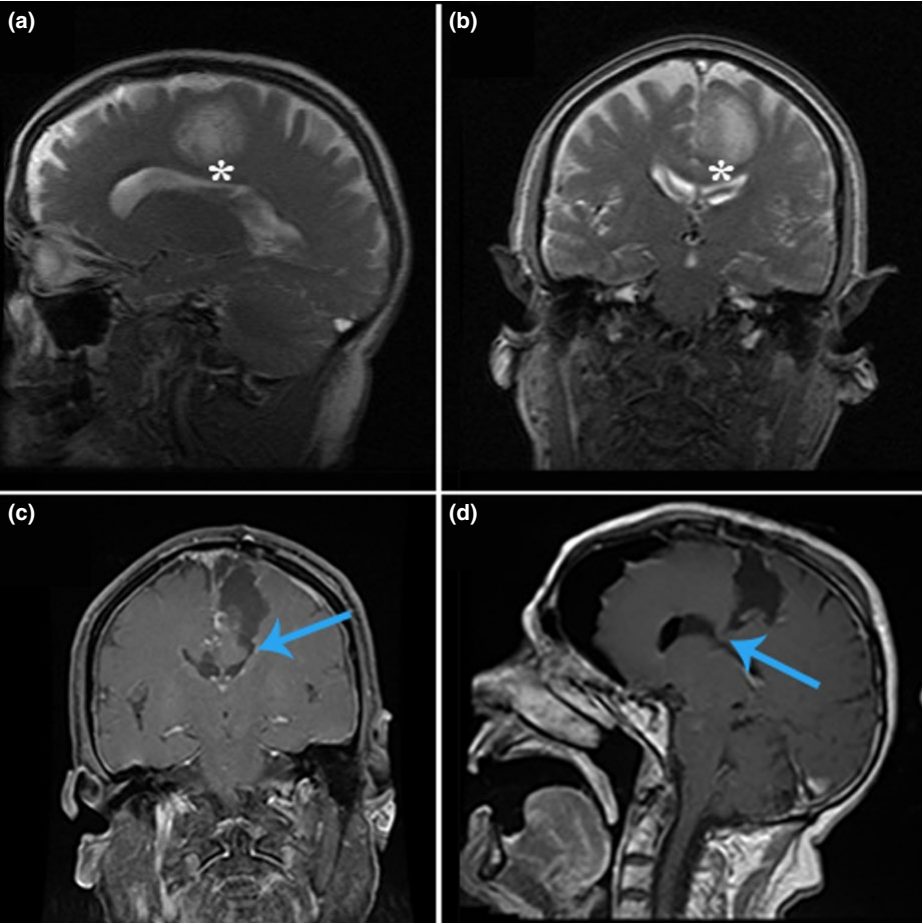
Permanent supplementary motor area syndrome, case 2. Hyperintensities seen on T2 sagittal (a) and coronal (b) imaging demonstrating a patient with glioblastoma. As in Figure 2, postoperative images of T1 with contrast enhancement showing dissection into the corpus callosum (CC) from coronal (c) and sagittal (d) views (blue arrows). Asterisks in preoperative images of (a) and (b) designate CC location

A 77‐year‐old man presented with a 2‐week history of progressive right‐sided weakness. He was found to have a left frontoparietal mass on magnetic resonance imaging. One day later, he underwent a nonawake craniotomy for tumor resection. Pathology revealed glioblastoma. Postoperatively, the patient demonstrated worsening right‐sided hemiparesis as well as mutism. The weakness has not improved on long‐term follow‐up. He also remains largely averbal. Preoperative and postoperative imaging is shown in Figure 3.

### 3.3 Commonalities between the cases

**Figure 4:**
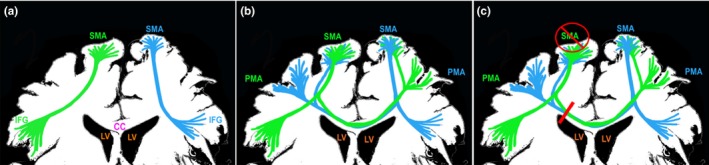
Simplified illustration of the frontal aslant tract (FAT) and newly described transcollasal projections of the FAT. (a) FAT connecting the supplementary motor area (SMA) and inferior frontal gyrus (IFG). Right FAT shown in green and left FAT in blue. (b) Transcollasal FAT fibers originate from the previously described FAT. The fiber bundles transverse the corpus callosum (CC) connecting controlatereal premotor areas. (c) Proposed mechanism of permanent SMA syndrome. Dissection into the CC with complete separation of the SMA from the contralateral hemisphere may result in permanent SMA syndrome

After a second patient did not recover from SMA syndrome on long‐term follow‐up, we began to investigate the mechanism of SMA recovery. Detailed analysis of postoperative images revealed that in these two cases, the CC fibers had been severed upon entering the ventricle. This was not noted in previous cases in which patients recovered. This led us to hypothesize the existence of bilateral connections between the motor planning areas. We then used tractography to analyze these regions. In all the cases we studied, in addition to the known bilateral connections between the SMAs, there appeared to be tracts from the FAT through the CC to the contralateral PMAs. These tracts joined and paralleled the ipsilateral FAT. Hence, we refer to this tract as the “crossed FAT.” A schematic of the FAT, crossed FAT fibers and our hypothesis of injury resulting in permanent SMA syndrome are given in Figure 4.

## 4 DISCUSSION

We describe a path of connectivity between the FAT and contralateral PMA through the CC. We believe this tract is a previously unrecognized component of the FAT and thus have termed this tract the “crossed FAT.” Based on our clinical experience, we suggest that this tract is important for recovery of the SMA syndrome in patients with SMA resection. There have been descriptions of SMA recovery utilizing an interhemispheric dependence but to our knowledge, no previous study has suggested a precise mechanism by which this occurs (Otten et al., 2012; Vassal et al., 2017). We provide this information with the hope that knowledge of this tract will improve surgical outcomes for patients with tumors in the SMA.

### 4.1 Connections of the SMA

Others have described white matter connections of the SMA (Lemaire et al., 2013; Vergani et al., 2014). The callosal connections defined by Vergani et al. were based on findings from anatomical and diffusion tensor imaging studies. These fibers originated in the SMA and ended in the contralateral SMA; however, there was no mention of fibers connecting to the contralateral PMA. Lemaire et al. also described a transcallosal SMA fascicle but made no mention of the PMA (Lemaire et al., 2013). Neither of these studies described the transcallosal path in relation to the FAT nor made an association with the tract to SMA syndrome.

Retrograde tracer studies on nonhuman primates have found that the majority of callosal inputs to the pre‐SMA and SMA‐proper originate from the contralateral SMA and contralateral PMA (Liu, Morel, Wannier, & Rouiller, 2002). In particular, one study of four different macaques found that neuronal projections from the contralateral PMAs accounted for 20.3%–29.3% of all projections to these SMA regions (Liu et al., 2002). SMA studies with diffusion tensor imaging have also shown distinct connections from SMA and pre‐SMA to the ipsilateral and contralateral striatum (Lehericy et al., 2004). Similar connections have been confirmed in primates with anterograde tracing (McGuire, Bates, & Goldman‐Rakic, 1991). Connections to the striatum are relevant as studies in stroke patients suggest that lesions of white matter tracts from SMA to striatum may be implicated with speech recovery in patients with aphasia (Naeser & Palumbo, 1994; Naeser, Palumbo, Helm‐Estabrooks, Stiassny‐Eder, & Albert, 1989). In accordance with the stroke studies, there is a case example of a patient with aphasia who does not recover speech despite having a functionally active left SMA (Martin et al., 2009). The patient is found to have damage to the white matter pathways projecting from the SMA. This case example and other studies on SMA connectivity illustrate the importance of peripheral SMA connections.

We confirmed SMA and contralateral PMA connections with diffusion tractography and gross anatomical dissection. It is difficult to differentiate antiparallel fibers in the CC with gross anatomical dissection. However, with GQI‐based tractography, the tracts can be separated based on a region of interest, allowing us to differentiate the hemisphere from which the tract originated. General morphology of the major tracts was consistent between these modalities, similar to other authors (Catani et al., 2012). Furthermore, all 10 of the cadaveric brain dissections demonstrated these tracts consistently. It should be noted here that one of the primary motivations for performing cadaveric brain dissections was to tie the tractography results to ground truth as a major confounding factor in using deterministic tractography programs is the possibility that the algorithm will generate artefactual tracts.

### 4.2 Proposed mechanisms of SMA recovery

Recovery from SMA syndrome is well documented, yet our knowledge of how this occurs is still limited (Abel, Buckley, Morton, Gabikian, & Silbergeld, 2015; Nakajima et al., 2015; Vassal et al., 2017). Among the mechanisms suggested are simpler explanations of local ischemia, retraction injury and cerebral edema responsible for transient deficits, to the more complex, involving local and distant compensatory sites (Rosenberg et al., 2010; Vassal et al., 2017). The explanations that attribute transient deficits to postoperative irritation and edema do not correlate with recovery time seen in other brain regions (Abel et al., 2015). However, the ability of the brain to reorganize functions to new sites has been studied extensively in its role in language and motor preservation (Kielar, Deschamps, Jokel, & Meltzer, 2016).

Functional connectivity studies support the idea of compensation from the contralateral hemisphere (Ogawa et al., 1993). Multiple fMRI studies have revealed an increase in activity of the nonlesioned SMA compared to baseline values after ischemia, tumor invasion, and surgical operations (Acioly, Cunha, Parise, Rodrigues, & Tovar‐Moll, 2015; Krainik et al., 2004; Naeser et al., 2004; Otten et al., 2012; Walther et al., 2009). Increased activity of the healthy, contralateral hemisphere has also been associated with less weakness in studies of brain tumors that involve motor networks (Otten et al., 2012). Brain tumor patients with normal strength had increased activity on resting MRI between homologous motor structures (SMA and premotor cortex) as compared to brain tumor patients with weakness. fMRI activity was also increased compared to that of control patients without brain tumor or weakness (Otten et al., 2012). In persons with SMA syndrome, increased activity in the nonlesioned hemisphere, including PMAs, has been associated with decreased recovery time (Krainik et al., 2004). Evidence from these studies suggests that SMA recovery occurs through a compensatory mechanism involving the contralateral hemisphere.

Earlier studies have shown that increases in postoperative fMRI activation in regions near tumor occurrence are related to SMA syndrome recovery. Rosenburg et al. used intraoperative direct cortical stimulation (DCS) and fMRI to assess compensation in other cerebral regions when DCS was applied to the SMA. They found that greater activity on fMRI adjacent to the disrupted SMA was correlated with retaining functional tasks (Rosenberg et al., 2010). They did not find that increased interhemispheric activity was correlated to retaining functional tasks. However, as they point out, their study only assessed short‐term functional deficit compensation and was unable to examine neural correlates related to long‐term recovery processes. Thus, changes in interhemispheric connectivity during the recovery phase of SMA syndrome are not well understood.

### 4.3 Implications for cerebral surgery

Gliomas in the frontoparietal region commonly necessitate partial or total SMA resection and are the most common cause of SMA syndrome (Duffau & Capelle, 2004). The SMA syndrome was first described by Laplane, et al. (1977) and involves a transient contralateral motor deficit. With involvement of the dominant SMA, the patient may also experience speech impairment (Abel et al., 2015; Bannur & Rajshekhar, 2000; Tate et al., 2011; Vergani et al., 2014). The severity of motor deficits can range from mild hypokinesis to severe hemiparesis. Most patients recover within weeks to months with initial deficit severity having no correlation with recovery time (Abel et al., 2015).

It is possible that some of the WM fibers from the pre‐SMA were included in the dissection and tractography analysis of the SMA. As previously mentioned, the SMA and pre‐SMA have close proximity, and the border that separates these structures is relatively ambiguous when compared to the divisions of other brain regions. Furthermore, Catani et al. described the FAT as having projections to both SMA and pre‐SMA (Catani et al., 2012). We acknowledge the possibility of damage to pre‐SMA fibers in the patient who experienced complete hemiplegia and mutism. However, based on neurosurgical documentation, we believe the SMA was the primary site of insult.

We cannot conclude with certainty that preservation of the crossed FAT fibers will prevent development of long‐term SMA syndrome. There are other mechanisms by which these deficits could occur. It is possible that the patients inability to recover was a result of SMA resection and not from a CC lesion. Although, based on our operative experience, we do not believe this to be the case. Other patients who have had similar SMA resections without insult to the CC recover relatively well. It is also possible that damage to the CC is interfering with homologous connectivity between right and left SMA or that it is a combination of the disruption of the homologous and nonhomologous connections causing permanent deficits.

We suggest that preservation of the crossed FAT may be necessary for recovery from SMA syndrome in some patients. Without cellular electrophysiological recording to reveal bihemispheric communication, one cannot definitively conclude that fMRI demonstrates true compensation. These experiments would be limited by ethical considerations in humans and by the differences in human and nonhuman SMA syndrome presentation found in nonhuman primates (Potgieser et al., 2014).

## 5 CONCLUSION

Although most patients with SMA syndrome experience symptomatic resolution, some do not. Preservation of particular white matter pathways is likely to be essential in determining recovery. We describe a white matter tract that passes through the CC to connect the PMA to the contralateral SMA and PMA, providing a possible mechanism for recovery from SMA syndrome.
